# Synchronization dynamics and evidence for a repertoire of network states in resting EEG

**DOI:** 10.3389/fncom.2012.00074

**Published:** 2012-09-28

**Authors:** Richard F. Betzel, Molly A. Erickson, Malene Abell, Brian F. O'Donnell, William P. Hetrick, Olaf Sporns

**Affiliations:** ^1^Department of Psychological and Brain Sciences, Indiana UniversityBloomington, IN, USA; ^2^Department of Psychiatry, Indiana University School of MedicineIndianapolis, IN, USA; ^3^Larue D. Carter Memorial HospitalIndianapolis, IN, USA

**Keywords:** EEG, microstates, networks, dynamics, resting-state

## Abstract

Intrinsically driven neural activity generated at rest exhibits complex spatiotemporal dynamics characterized by patterns of synchronization across distant brain regions. Mounting evidence suggests that these patterns exhibit fluctuations and nonstationarity at multiple time scales. Resting-state electroencephalographic (EEG) recordings were examined in 12 young adults for changes in synchronization patterns on a fast time scale in the range of tens to hundreds of milliseconds. Results revealed that EEG dynamics continuously underwent rapid transitions between intermittently stable states. Numerous approximate recurrences of states were observed within single recording epochs, across different epochs separated by longer times, and between participants. For broadband (4–30 Hz) data, a majority of states could be grouped into three families, suggesting the existence of a limited repertoire of core states that is continually revisited and shared across participants. Our results document the existence of fast synchronization dynamics iterating amongst a small set of core networks in the resting brain, complementing earlier findings of nonstationary dynamics in electromagnetic recordings and transient EEG microstates.

## Introduction

A growing body of evidence from electromagnetic recordings and neuroimaging suggests that human cognition arises from patterns of neural activity unfolding across the brain (Mesulam, 1990; Bressler, 1995; McIntosh, 2000; Bressler and Kelso, 2001; Varela et al., 2001). These patterns form functionally distinct neurocognitive networks that are ultimately shaped and coordinated by the brain's anatomical connections (Bullmore and Sporns, 2009; Bressler and Menon, 2010). The fluidity of human cognition is thought to be reflected in the temporal dynamics of these networks, involving transient and metastable coordination (Kelso, 1995; Friston, 1997, 2000) that manifests itself in the continual formation and dissolution of synchronized neural activity on short time scales in the range of tens to hundreds of milliseconds. In this report, fast temporal dynamics were characterized in electroencephalographic (EEG) recordings of the human brain at rest, by combining highly temporally resolved measurement of whole-brain synchronization with network analysis.

Neuronal time series recorded from sensors (e.g., EEG) or brain voxels (e.g., fMRI) can be rendered into functional brain networks (Stam and Rejneveld, 2007; Bullmore and Sporns, 2009). Such networks consist of nodes (corresponding to recording sites or brain regions) linked by pair-wise estimates of dynamic coupling, generally a linear or nonlinear measure of statistical dependence expressing correlation or coherence. Recent work employing graph theoretic analysis has focused on characterizing the topology of static (temporally invariant) networks, for example of anatomical connections (Hagmann et al., 2008), or of long-time averages of neural dynamics, for example those observed during spontaneous neural activity (Wang et al., 2010). Considerably less is known about the dynamics of brain networks over time. Building on earlier studies of the structure of dynamic EEG patterns (Lehmann and Skrandies, 1980; Lehmann, 1990), closer examination of neural time courses and their synchronization dynamics has recently revealed fluctuations and nonstationary transitions in functional brain networks on multiple time scales. Multistability, time-varying network topology and nonstationary dynamics have been detected in EEG (Freyer et al., 2009; Latchoumane and Jeong, 2010; Chu et al., 2012), electrocorticography (ECoG; Kramer et al., 2010) and magnetoencephalography (MEG) recordings (de Pasquale et al., 2010), respectively. Slower changes in functional networks also occur in spontaneous fluctuations of the BOLD response measured with fMRI (Chang and Glover, 2010; Smith et al., 2012).

The present goal was to characterize the dynamics of whole-brain functional networks at high temporal resolution by monitoring the changing topology of these networks across time. It was predicted based on prior work that patterns of synchronization would undergo rapid fluctuations resulting in continual reconfigurations of functional brain networks. As predicted, fast (~100 ms) dynamics of whole-brain synchronization were observed during resting-state EEG. Transient episodes of stability corresponded to slow-changing network topology, while intermittent transitions were associated with rapid changes in synchronization. The resulting changes in network structure defined coherent states, and upon aggregating over many recording epochs and all participants, revealed a small repertoire of network states that was continually revisited over time.

## Methods

**Figure 1:**
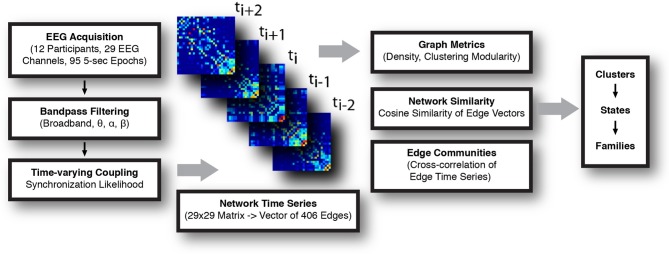
**Schematic for the construction and analysis of time-varying networks.** EEG signal was acquired, parsed into 5 s long epochs, and filtered. Synchronization likelihood (SL) was calculated for each pair of channels in the frequency band of interest, and functional networks (electrode × electrode) constructed at each time step. Analysis of the network time series consisted of calculating graph metrics at each instant, assessing network similarity, and detection of sets of highly correlated edge time series (edge communities). Temporally invariant network states were defined, first, by representing each network as a vector and calculating the normalized cosine distance between all networks. These distances were represented in a similarity matrix, which was subsequently clustered. Series of temporally adjacent networks assigned to the same cluster were taken to comprise a coherent network state. State families were determined by assessing the extent to which similar network states recurred across all recording epochs.

A summary of the data processing pipeline is shown in Figure 1.

### Participants

After obtaining informed consent in accordance with our institutional guidelines, 12 healthy adults participated in the present study (five male, seven female; mean age = 41.67 years; SD = 13.61 years). Each participant was administered the Structured Clinical Interview for the DSM—Non-Patient Edition (SCID-NP; First et al., 2002) to rule out the presence of Axis I mental disorders.

### Electroencephalogram recording

Electroencephalogram (EEG) was recorded from 29 cortical Ag-AgCl electrodes (International 10–20 cap system; Falk Minow Services/EasyCap, Munich, Germany) at 1000 samples per second with a NeuroScan, Inc. (El Paso, TX) bioamplification system. EEG data were filtered online with an analog band-pass filter with a low cutoff of 0.02 Hz and a high cutoff of 200 Hz and gain of 10 K. Vertical and horizontal electrooculogram (EOG) was also recorded. EEG data were recorded using the NeuroScan Aquire 4.1 software package and impedances were kept below 10 kΩ for all electrode sites. Participants were seated in an electrically and acoustically shielded chamber and instructed to remain alert with their eyes closed while EEG was recorded continuously for approximately 5 min.

### Signal processing

Continuous EEG data were divided into 5 s epochs and down-sampled to 500 Hz (2500 discrete time points). Conservative artifact rejection was performed using algorithms in the Matlab® (Version 2011a, The Mathworks, Natick, MA) toolbox EEGLab (Delorme and Makeig, 2004) as well as by visual inspection. Any epochs containing artifacts defined by (1) microvolts exceeding a value of ±200, (2) slopes larger than 75 μ V, and/or (3) abnormal distributions of μ V values were rejected from analysis. In this context, an abnormal distribution is characterized as any channel exhibiting an absolute μ V value more than three standard deviations from the channel mean or abnormally high or low kurtotic values of μ V distribution. Afterward, a visual inspection was performed on the remaining data to reject any epochs that contained undetected artifacts. All 12 subjects retained at least two artifact-free epochs to be included in the analysis (*N*_*epochs*_ = 7.92 ± 2.91). Epochs were filtered to yield signals in four frequency bands of interest: broadband (4–30 Hz), theta (θ; 4–8 Hz), alpha (α; 8–13 Hz), and beta (β; 13–30 Hz). The EEG montage was referenced to the average signal rather than a single electrode. This reduced the likelihood that spurious synchronization was detected due to contributions from a single reference electrode (e.g., nasal electrode). Each channel was standardized to have zero mean and unit variance. It should be noted that EEG is sometimes references to other electrodes and that this choice of reference has effects that propagate to measures of coupling (Nunez et al., 1997).

### Synchronization likelihood

Synchronization likelihood (SL) is a measure for detecting non-linear, dynamical coupling between pairs of recording series (Stam and van Dijk, 2002; Altenburg et al., 2003). The process for computing SL is straightforward. Suppose EEG recording series, *x*^*k*^_*i*_ = *x*^k^_1_ … *x*^*k*^_*N*_, where *k* = 1 … *K* and *i* = 1 … *N* represent channel and sample indices, respectively.

The state of each recording series at sample *i* is defined by the by the vector, ***X***^*k*^_*i*_ constructed through a time-delay embedding operation:

$$X_{i}^{k}\text{=}\langle x_{i}^{k}\;x_{i\text{+}l}^{k}\;x_{i\text{+2}l}^{k}\cdots x_{i\text{+}\left(m\text{-3}\right)l\;}^{k}\;x_{i\text{+}\left(m\text{-2}\right)l\;}^{k}\;x_{i\text{+}\left(m\text{-1}\right)l}^{k}\rangle$$

Here, the parameters *l* and *m* are the lag and embedding dimension, respectively. Appropriate values for these parameters and others are discussed in the next section.

At each sample *i*, we compute the auto-recurrence of reference vectors ***X***^*k*^_*i*_. We define a recurrence by, first, computing for each channel the Euclidean distance from the reference vector to all ***X***^*k*^_*j*_, where all *j* satisfies the inequality *w*_2_ > |*i* − *j*| > *w*_1_:

$$D_{j}^{k}\text{=}\left\|X_{i}^{k}\text{-}X_{j}^{k}\right\|$$

The indices of the *n*_rec_ smallest distances are defined as recurrences and stored in the vector, ***J***^*k*^. The SL then, is defined at sample *i* for any two channels, *k*_1_ and *k*_2_, as follows:

$$S_{i}^{k_{\text{1}},k_{\text{2}}}\text{=}\frac{\left|J^{k_{\text{1}}}\cap J^{k_{\text{2}}}\right|}{n_{\text{rec}}}$$

Succinctly, SL enumerates the number of times two reference vectors recur simultaneously out of the total possible number of recurrences.

To compute SL, we must specify values for the parameters *l*, *m*, *w*_1_, *w*_2_, and *n*_rec_. Fortunately, these parameters are constrained by the frequency content of the EEG series, *x*^*k*^_*i*_ (Montez et al., 2006). Lag, *l*, and embedding dimension, *m*, are chosen according to the fastest and slowest oscillations in the recording series. Lag must sample the fastest component no fewer than twice per cycle in order to accurately reconstruct the series' dynamics, in accordance with the Nyquist sampling theorem. The embedding dimension specifies the dimensionality of the state vectors ***X***^*k*^_*i*_ and is chosen to ensure that at least one whole cycle of the slowest oscillations are captured, as well.

The parameters *w*_1_ and *w*_2_ collectively specify the size of the window in which recurrences may take place. The window's leading edge, *w*_1_, is set large enough to ensure that putative recurrences are due not to auto-correlated states. The window's tailing edge, *w*_2_, is selected in conjunction with the maximum number of recurrences, *n*_rec_. Together, *w*_2_ and *n*_rec_ control the “recurrence density,” or the number of recurrences per window, *w*_2_ − *w*_1_.

In the present study, we computed SL between all pairs of channels and at each sample for each frequency band of interest. Parameters for this computation were selected according to the procedure described above. For the broadband, the fastest oscillatory component is ≈ 30 Hz while the slowest is ≈ 4 Hz. In this case, the lag parameter, *l*, and embedding dimension, *m*, were set to 5 and 24 samples, respectively. Together, these parameters specify a state vector spanning (*m* − 1)*l*, or 230 ms. The window's leading edge was set to two times this length, *w*_1_ = 460 ms, accordingly. *n*_rec_ was fixed at 10 and *w*_2_ = 858 ms.

It should be emphasized that the constraints on these parameters discussed above are imposed only for practical reasons related to the frequency content of the EEG recordings. In general, these parameters can assume a much broader range of values. For instance, time-delay embedding operations can be performed with any arbitrary lag, *l*, provided that the embedding dimension, *m*, is at least twice the dimension of the underlying attractor (Takens, 1981).

### SL networks and graph measures

SL estimation resulted in a three-dimensional array, ***S***^*k*_1_,*k*_2_^_i_, with one dimension, *i*, corresponding to observations or sample points and the remaining two dimensions, *k*_1_ and *k*_2_, corresponding to channel number. “Slicing” the array at any discrete time point yielded a symmetric, channel-by-channel matrix, whose 29×29 elements represented a network of pair-wise estimates of SL between two channels, or equivalently, after discarding the lower triangle of the 29×29 matrix, a 406-element SL vector, ***S***^*e*^_*i*_, *e* = 1 … 406. Each of these 406 elements represents a network edge linking two nodes (electrodes).

To assess whether the finite (non-zero) estimate of synchronization obtained from electrode pairs was merely due to stochastic fluctuations of a stationary, multivariate linear process, we first generated a null distribution of edge weights from an ensemble of phase-randomized surrogate data. Excursions in the data of SL values outside of the distribution derived from this ensemble argue for a rejection of the null in favor of a more complex underlying process. Briefly, this procedure entailed Gaussian resampling each EEG channel and Fourier transforming the resampled series. Random phase was added to all channels uniformly so that the additional phase for channels *i* and *j* is identical, and then the series was inverse transformed and rescaled by an inversion of the Gaussian resampling (Theiler et al., 1992). These surrogates possessed the same mean, standard deviation, and approximate auto-correlation structure as the empirical data. 10,000 such surrogates were generated for each epoch and SL computed. The aggregated SL values for each edge at each sample, then, served as null distributions.

A non-parametric test of the exact hypothesis (one-tailed Wilcoxon signed-rank test) was used to generate *p*-values for each edge. For the broadband, this procedure means computing 406 edges × 1529 samples = 620,774 tests. A typical *p*-value cutoff of *p* < 0.05 would mean that we would expect about 31,000 edges being falsely declared present over the course of the entire epoch. To control the false discovery rate (FDR) we used a linear step-up technique with *q* < 0.0001 to compute adjusted *p*-values for the edges at each sample point (Benjamini and Hochberg, 1995). From the results of these tests, we retained empirical edge weights whose *p*-values were less than or equal to the adjusted *p*-value. Otherwise, the edge weight was set equal to zero.

Weighted and undirected SL networks obtained from a single time point or averaged over entire epochs were analyzed with methods from graph theory, using the Brain Connectivity Toolbox (www.brain-connectivity-toolbox.net; Rubinov and Sporns, 2010). The density of the network, which corresponds to the average node strength, was derived as the global average of all matrix elements. The clustering coefficient, expressing the degree to which highly synchronized node pairs are synchronized with common partners, was computed for each SL vector, followed by normalization to a population of randomized control networks with equal degree distribution. The modularity was computed using a weighted version of a graph-based metric (Girvan and Newman, 2002) after identifying the optimal module partition.

### Assessment of network similarity and of edge communities

The time series of SL vectors describing coherent synchronization patterns allowed the assessment of network similarity over time, as well as the extraction of sets of temporally co-varying network edges forming “edge communities.” Network similarity was examined along the time dimension by tracking the persistence and recurrence of global SL patterns. A similarity matrix was generated for each recording epoch by calculating the normalized cosine similarity between all pairs of SL vectors. These similarity matrices formed the basis for the identification of sequences of coherent states (block structure along the diagonal) as well as their approximate recurrences (“hot” off-diagonal patches). Edge communities were detected by computing the cross-correlation among all 406 edge time series and clustering of the resulting correlation matrix.

### Evolutionary clustering

We defined a network state as a set of highly similar, temporally contiguous SL vectors. To detect the boundaries of such states, we sought to partition the set of all SL vectors belonging to each epoch into non-overlapping clusters.

Here, we define a partition as a function mapping SL vectors to clusters, so that for any SL vector, it has a unique cluster assignment. The “goodness” of a given partition is scored by a quality function, in this case, a variation of the common Dunn's index (Dunn, 1973). In general, Dunn's index rewards partitions whose clusters simultaneously possess maximal within-cluster homogeneity and between-cluster heterogeneity. As measures of within-cluster homogeneity and between-cluster heterogeneity we used the average Euclidean distance of data from their respective cluster centroid and the Euclidean distance between centroids, respectively.

Determining the partition that maximizes Dunn's index, however, is an NP-complete problem. As such, a number of heuristics have been proposed that generate good (but sub-optimal) partitions, the two most prevalent being k-means clustering and variations of agglomerative hierarchical clustering. In this report, we use neither of these approaches, adopting, instead, an evolutionary heuristic.

Evolutionary heuristics are algorithms inspired by the tenets of natural selection, namely that the most-fit individuals tend to reproduce, propagating their genome to subsequent generations. An evolutionary clustering algorithm, then, behaves in the following way: At generation zero, a population comprised of random partitions is generated and each partition belonging to this population is assigned a fitness based on its goodness (e.g., Dunn's index). A new population is generated by selecting members of the previous population based on their fitness scores, with a bias toward accepting better-fit individuals. Before the new population advance, however, a fraction of its members are perturbed by a one or more evolutionary operators, the most common of which are the mutation operator and crossover operator. The mutation operator mimics random genetic alterations that sometimes take place in natural populations. In the context of an evolutionary clustering algorithm where each individual is a partition of some dataset, the mutation operator operates on a single individual, altering the datum-to-cluster mapping in some minor way. The crossover operator, on the other hand, mimics reproduction and the exchange of genetic material between two parents to create an offspring. In an evolutionary clustering algorithm, this operator works by randomly selecting two partitions and exchanging a fraction of their datum-to-cluster mappings. These operators (and others like them) drive the search for the optimal partition in the space of all possible partitions by continually generating new partitions.

There are two main reasons for adopting an evolutionary approach for clustering rather than a more traditional k-means or hierarchical approach. The more traditional approaches require that you specify the number of clusters into which data are partitioned. This is not ideal, because in many cases the number of clusters is unknown, *a priori*. The evolutionary approach, by exploring the space of possible solutions and ranking their fitness, naturally progresses toward a partition with high fitness irrespective of the number of clusters into which it assigns a given dataset. The second reason for adopting an evolutionary approach is that it avoids the determinism of hierarchical clustering. Hierarchical clustering begins by assigning each datum to its own cluster and successively merging, or agglomerating, the clusters nearest one another, where proximity is determined by a distance metric and a linkage function, which specifies for the rule for merging two clusters. Because the same linkage function is applied for the entire agglomerative process and the mergers it specifies do not necessarily lead to better fitness, the partition returned by this process is likely far from optimal. Evolutionary approaches, in contrast, are driven toward partitions with high fitness, since there is a greater chance that highly fit partitions survive from one generation to the next.

In this report, we expand upon this evolutionary heuristic in the following ways: Our initial population of partitions (*N* = 50) was not generated at random; instead we seeded our initial population with partitions generated by hierarchical clustering runs using random cluster numbers and linkage functions. The generation of a single member of population begins by selecting randomly cluster numbers from 2 to 100. In this manuscript, we selected 25 cluster numbers without replacement. For each cluster number, we hierarchically cluster our data using single, average, and complete linkage functions. Each clustering generates a full partition. That is, each datum is assigned a cluster. Population members, then, are seeded by randomly choosing clusters from this set of partitions and assigning, in the population member, the corresponding data to their own cluster. This step is repeated until each datum in the population member has an assignment.

We also introduced a “jitter” evolutionary operator. This operator can be viewed as a more targeted version of the mutation operator, exploiting the temporal dependencies of the network states. The jitter operator acts on a single cluster for given partition, isolating within that cluster a sub-cluster comprised of two or more temporally contiguous SL vectors. The time points at which these SL vectors are observed within the epoch are noted, and one of three operations are performed: (1) the sub-cluster is expanded by adding to the cluster the SL vectors immediately preceding and following it; (2) the sub-cluster is contracted by removing the outermost SL vectors belonging to the sub-cluster and assigning them to new clusters; or (3) expanding at one boundary while contracting at the other, effectively shifting the sub-cluster one sample point either forward or backward in time.

With each passing generation, a small fraction of the population was replaced at by new, randomly-generated partitions constructed in the exact manner as the initial population. These random partitions ensure that the population has a constant influx of new partitions that can be used by other operators (e.g., crossover).

Lastly, we retained the five most-fit individuals from the previous generation without alterations. The evolutionary clustering algorithm was run for 1500 generations and returned, in the end, the partition maximizing Dunn's Index.

SL vectors were placed into clusters without considering their time of occurrence within the epoch. The assignment of each SL vector to a similarity-based cluster allowed the identification of coherent network states that were separated by state boundaries. These boundaries were defined as time points where SL vectors switched from one cluster to another. Since network states could recur in a single epoch, the number of states often exceeded the number of clusters. To represent each network state, a single SL vector was constructed by averaging over the SL vectors belonging to a contiguous cluster. The length of each contiguous cluster defined the duration of its network state. For each frequency band, the collection of durations for all states was aggregated and probability mass functions were estimated.

### Network state repertoire

The number of clean epochs was variable among participants. As a consequence, many subjects were over- or under-represented in terms of number of network states. To correct for this sampling bias and to characterize the repertoire of network states, we constructed 100 ensembles of network states. Each ensemble was constructed in the following way: For a given frequency band, we found the subject whose total contribution of network states accounted for the smallest duration of time and divided this time by four. This time was set as the target time for that frequency band. To generate a single ensemble, then, we sampled states randomly from each subject until the total duration of the sampled states of that subject exceeded the target time. Each ensemble, then, contained states for each subject such that the total duration of these states was approximately equal across all subjects. The cosine similarity between all pairs of networks was constructed identically to the method described earlier. The dimensionality and density of the vector representation of networks implies that two uncorrelated network states might be given a high similarity score simply by chance. We sought to extract pairs of network states whose similarity score was greater than chance levels. To do this, we constructed a null distribution of similarity scores by randomly permuting each network's edges and computing a new similarity matrix. This process was repeated 1000 times so that for every pair of networks in any ensemble, a distribution of similarity scores that served as the null hypothesis to those networks' empirical similarity score was generated. The significance of any similarity score was computed using a one-sample, one-tailed *t*-test with *p* < 0.000001. A binary reconstruction of the similarity matrix, where each cell was {1} if the similarity score was larger than expected by chance and {0} otherwise, was then clustered by optimizing the modularity valuation index using a greedy algorithm (Blondel et al., 2008). The resulting partition organized the network states in each ensemble into families of states. Here, a family is defined as a community in the modularity maximization step. The number and relative size of state families were largely consistent across ensembles. From these, an average partition was generated by aggregating families and once again optimizing the modularity score of the family-by-family similarity matrix.

It should be mentioned that the above methods are somewhat demanding from a computational point of view. SL computation and clustering using an evolutionary approach are not the fastest means of estimating coupling between time series and clustering data, respectively. These methods were adopted for this project principally because SL is capable of detecting non-linear coupling between two systems and also does not require the systems to exhibit near oscillatory behavior which is necessary for coupling measures that depend on the relative phase of two signals. As alternatives to the evolutionary approach to clustering, we first employed well-known, widely available clustering techniques such as agglomerative hierarchical clustering and k-means clustering. These clustering algorithms were sensitive to free parameters and non-determinacy. They also generated partitions that tended to over- or under-cluster the networks. The evolutionary approach to clustering was ultimately adopted because it led to more stable partitions across runs and gave rise to higher quality partitions, as measured by Dunn's Index, that exceeded (and in some instances greatly exceeded) those from the other clustering methods.

## Results

**Figure 2:**
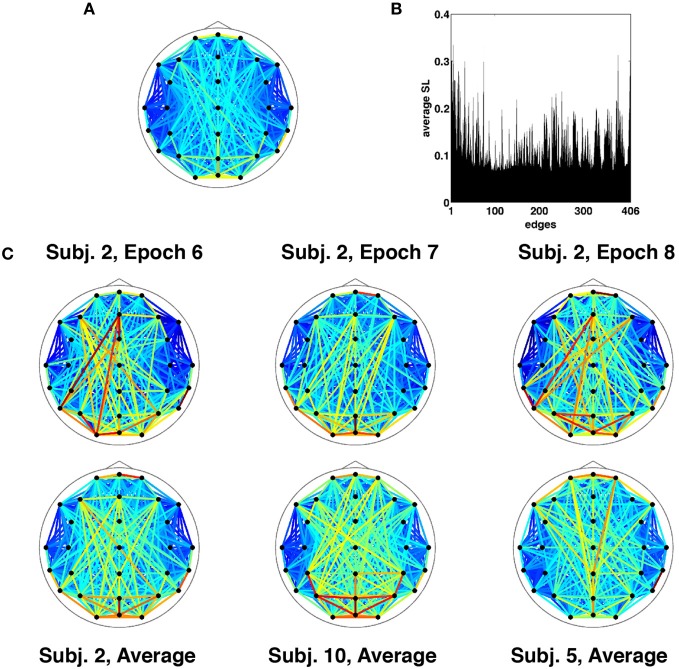
**Time-averaged SL. (A)** SL averaged across all recording epochs. Here and in every other topological map, the color of each edge denotes the magnitude of SL between the corresponding EEG electrodes. **(B)** SL averaged across a single recording epoch, revealing a number of edges that on average are highly synchronized for the duration of the recording. **(C)** Topographic representation of time-averaged SL for multiple recording epochs from the same participant exhibit similar patterns of synchronization (top row). The bottom row shows SL maps averaged across time and recording epochs for select participants.

Results are for the broadband frequency range (4–30 Hz), unless otherwise stated. Given the choice of SL parameters and the necessary removal of time points at the start and end for buffering, each epoch of broadband SL data consisted of 1529 discrete time points corresponding to 3.058 s (2 ms per time point). At each time point, the SL was measured between all 29 channels and represented as a vector of $406=\left(\begin{array}{c} 29\\ 2 \end{array}\right)$ unique relations or edges. Thus a single epoch consisted of 406 SL edge time series sampled over 1529 time points. The topographic pattern of edge synchronization measured by SL is depicted in Figure 2A, shown as a network whose edges represent global averages of SL over all time points from all epochs and all participants. Global averages of edge synchrony varied across edges (Figure 2B), with marked differences in the average synchrony across the network. Comparison of single epochs across time in a single participant as well as across participants showed highly similar SL topographic patterns indicating stability in long-time averages of global synchronization (Figure 2C). Some patterns characteristic for resting EEG (e.g., hemispheric symmetry, strong synchronization of frontal and occipital sensors) can be discerned.

**Figure 3:**
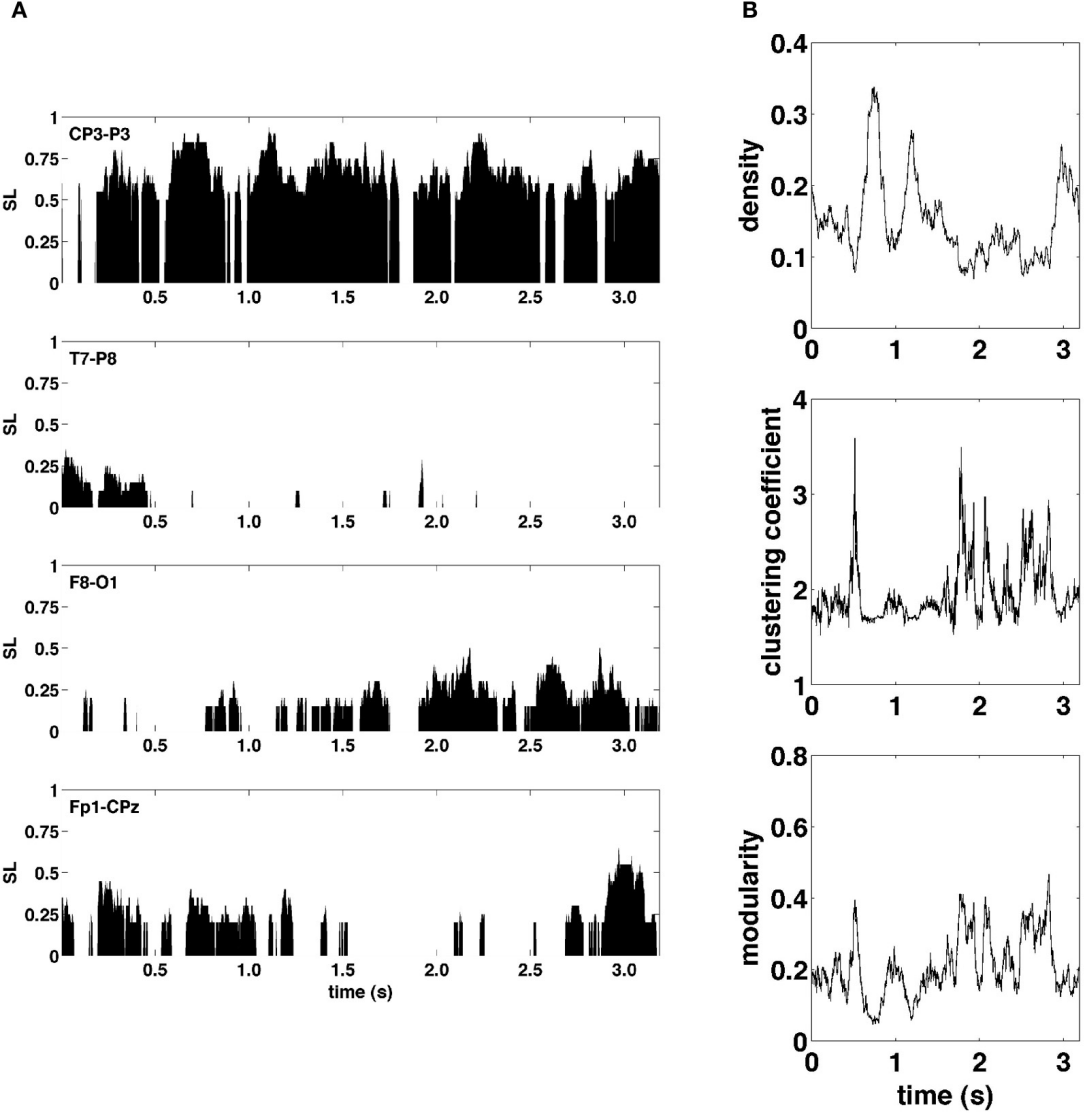
**Graph metrics and edge time series. (A)** Representative edge time series selected to highlight the varying degrees of synchronization and non-stationarity observed over the course of a single recording epoch. **(B)** Graph-theoretic metrics of density, clustering coefficient, and modularity also exhibit non-stationarity in their respective time series.

Figure 3A shows examples of SL time series for four representative electrode pairs (edges) from a single epoch. Clearly, these edge time series exhibited rapid fluctuations, varying in magnitude across the full range of possible synchronization levels. To assess how these edge dynamics influenced changes in network topology, global network measures for SL networks were computed at each time point. Figure 3B shows network measures as a function of time for the same epoch shown in Figure 3A. The global density of synchronized edges, as well as their network topology, indexed by clustering and modularity, exhibited significant fluctuations. Some fluctuations in topology were driven by fluctuations in network density, with sparser networks generally more highly clustered and modular (functionally segregated). Most notably, the community structure of SL networks showed rapid reconfigurations across the entire brain occurring on a time scale of tens to hundreds of milliseconds.

**Figure 4:**
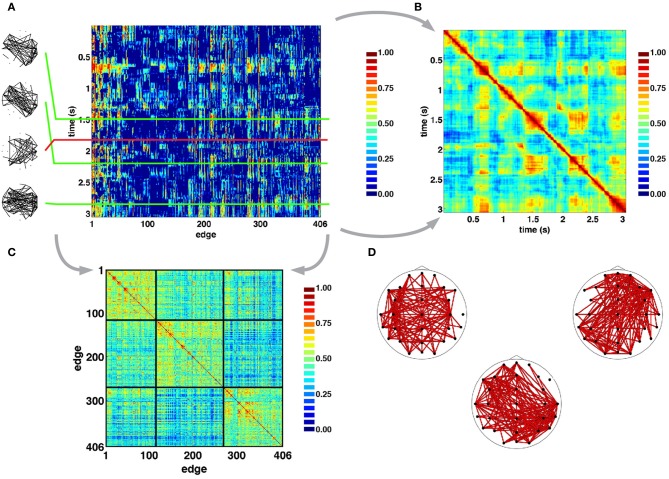
**Time series of SL networks. (A)** Each row in the SL matrix corresponds to an individual network (examples are shown in the inserts at the left). These networks exhibit periods of relative topological invariance (top two inserts), abrupt transitions (third insert from the top), and recurrences (bottom insert). **(B)** Normalized cosine similarity matrix between all pairs of SL vectors for the recording period shown in **A**. Hot colors represent pairs of highly similar networks, cool colors represent dissimilarity. The presence of block structure along the diagonal of the matrix suggest periods of quasi-stability and rapid intermittent transitions. “Hot” off-diagonal patches suggest recurrences of networks. **(C)**, Cross-correlation matrix of edge time series reordered to reveal clusters of edge communities, as detected in the epoch shown in panel A. **(D)**. Plots show topographic representations of edges constituent to the communities shown at the left. As such, each edge community is the set of edges whose time courses are strongly correlated with one another.

Figure 4A shows examples of SL networks at single time points (left panels), as well as time courses for all SL edges across a single representative epoch. This edges×time SL matrix was analyzed for temporal patterns of network similarity, as well as for groupings of network edges forming “edge communities.” To examine network similarity, a similarity matrix was constructed by computing the cosine distance across all 406-element SL vectors (Figure 4B). For pairs of SL networks that occurred close in time SL matrices consistently showed high similarity, and often gave the appearance of a “block structure” along the main diagonal, consistent with transient stability and intermittent instability of SL networks. Additionally, the off-diagonal portion of SL matrices showed instances of high similarity between SL networks that are separated by longer time intervals of up to several seconds. To identify edge communities, the cross-correlation of SL edge time series was computed and the resulting correlation matrix was subjected to modularity analysis (Figure 4C). For the epoch shown in Figure 4A, three edge communities consisting of edges that spanned both short and long distances across both hemispheres could be identified (Figure 4D).

**Figure 5:**
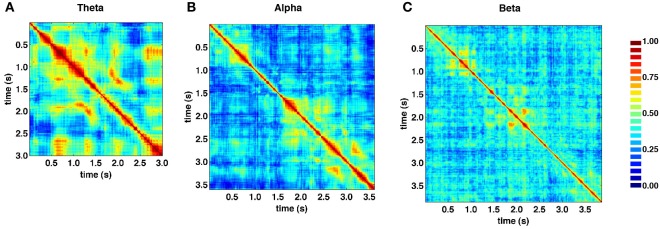
**Similarity matrices for specific frequency bands. (A)** Theta band (4–8 Hz). **(B)** Alpha band (8–13 Hz). **(C)** Beta band (13–30 Hz). SL exhibits strong frequency dependence, as well as time-dependent fluctuations across all bands, shown here for data from the same recording epoch depicted in Figure 4. In low frequency theta and alpha bands, much of the same structures visible in broadband similarity matrix (“block diagonal” and off-diagonal similarity) are present.

Figure 5 shows the similarity structure of SL networks obtained from the theta (4–8 Hz), alpha (8–13 Hz), and beta (13–30 Hz) frequency ranges, based on the same recording epoch depicted in Figure 4. While block-structure and hot off-diagonal patches are seen across all frequency bands, they are most pronounced at lower frequencies.

**Figure 6:**
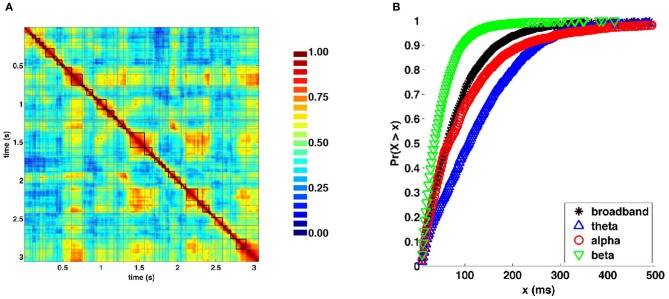
**Network states and durations. (A)** Representative similarity matrix from one recording epoch (compare with Figure 4B) with state boundaries overlaid. **(B)** Cumulative distributions of state durations (in milliseconds) aggregated across all recording epochs and frequency bands.

Visual inspection of the similarity structure (Figure 4B) is indicative of periods where SL networks remained coherent over time and exhibited little variation in topology, interspersed by rapid transitions. To objectively extract coherent states and state transitions, we implemented an evolutionary clustering algorithm for the classification of SL matrices for all recording epochs. Once clusters were extracted, changes in cluster membership across time were taken to correspond to changes in network states. Network states consisted of temporally coherent blocks of SL vectors. Within many of the epochs, clusters identified by evolutionary clustering contained two or more distinct instances of network states, corresponding to recurrences. Network states obtained from a representative epoch are shown in Figure 6A. Figure 6B shows cumulative distributions of the duration of these states, aggregated over all epochs and participants, and across frequency bands. The mean and median duration of network states were 82.4 and 64.0 (broadband), 129.1 and 108.0 (theta), 105.6 and 66.0 (alpha), and 48.6 and 38.0 ms (beta), respectively.

**Figure 7:**
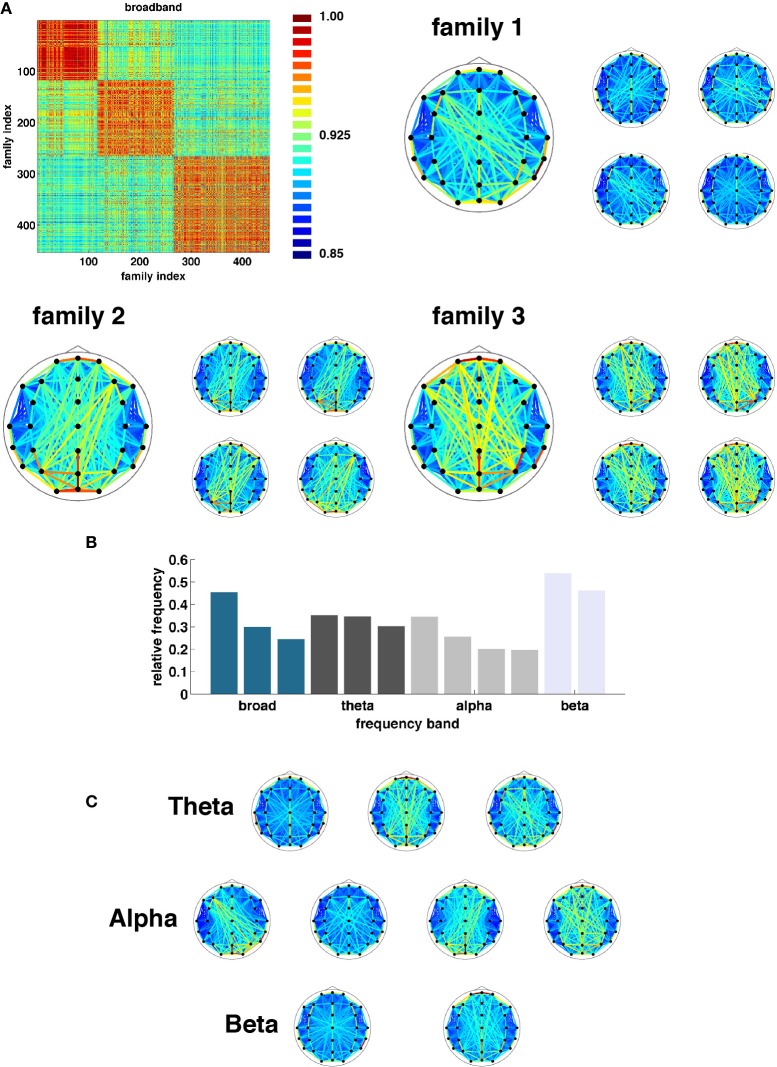
**Network families. (A)** Topographic representations of three broadband network families averaged over all constituent networks. To the right of each average representation are examples of network states assigned to each respective family. **(B)** Frequency at which each family of states was observed (ordered by band). **(C)** Topographic representations of average state family networks for theta, alpha, and beta frequency bands. Note similarity of network configurations across bands.

Once state boundaries were assigned, representative SL networks were constructed for each of the 3254 broadband states (2012, theta; 2927, alpha, and 7183, beta) identified across all 95 epochs. These states were obtained as a result of the evolutionary clustering step and are aggregated across all epochs. Once again, a similarity matrix was constructed based on the cosine distance between all pairs of network states. States in each frequency band were then partitioned into families based on similarity of their topological features (Figure 7). The number of families and the fraction of all states accounted for by each family varied across frequency bands. Broadband, for instance, was composed of three families accounting for 24.6, 30.0, and 45.4 percent of all observed states (Figure 7A). The other bands (theta, alpha, and beta) exhibited three (30.3, 35.1, and 34.6), four (19.6, 20.2, 25.6, and 34.6), and two (46.1 and 53.9) families (Figures 7B, C).

## Discussion

Fast changing and topologically coherent fluctuations in functional connectivity measured in resting state human EEG were identified by combining a nonlinear measure of synchronization (SL) with graph-theoretical network analysis. Fluctuations occurred on a time scale of tens to hundreds of milliseconds, involved spatially proximal and remote recording sites, and resulted in fast reconfigurations of network states. Network states recurred across time and formed a finite repertoire that was continually revisited. The findings were reported using primarily a broad frequency band (4–30 Hz), but similar findings were observed for narrow low frequency bands in the theta and alpha range.

The topology of functional networks obtained from synchronized brain dynamics in the resting-state and recorded with EEG and MEG has been shown to exhibit characteristic attributes, including dynamic synchronization (Breakspear et al., 2004) and small-world organization (Stam, 2004). Network topology is rapidly reconfigured between rest and task conditions (Bassett et al., 2006), varies with the level of task performance across individuals (Bassett et al., 2009) and changes across age (Micheloyannis et al., 2009). Some studies have suggested that network topology is disrupted in clinical conditions such as Alzheimer's disease (Stam et al., 2007) and schizophrenia (Micheloyannis et al., 2006; Rubinov et al., 2009).

The analysis of time-varying graphs is a relatively recent topic in network science, and is beginning to open up new avenues toward characterizing network dynamics (e.g., Tang et al., 2010; Grindrod et al., 2011). Relatively few studies so far have examined the time-dependence of brain functional networks in either rest or task conditions. Fallani et al. (2008) first introduced time-varying graph analysis of EEG data in the context of a simple motor task and reported temporally persistent edges as well as network motifs. Valencia et al. (2008) found evidence for time-dependent topology of functional brain networks by extracting event-related networks from electromagnetic time series. These topologies, while maintaining small-world architecture, showed characteristic changes on a time scale of hundreds of milliseconds that were associated with changes in task and stimulus condition. Dimitriadis et al. (2010) developed an approach for graph analysis of time-dependent networks derived from overlapping segments of EEG activity processed into series of adjacency matrices. Analysis of synchronization patterns in intracranial electrocorticogram recordings revealed significant changes in network topology during initiation, propagation, and termination of epileptic seizures (Kramer et al., 2010). Most recently, using resting-state EEG recordings Chu et al. (2012) have identified transient network configurations that rapidly converge onto a stable “template” or “core” network configuration when signals are averaged across time. Despite differences in the way networks were extracted from EEG time series, the topology of Chu et al.'s temporally stable core network has significant similarity to the network topologies reported here. Building on these studies, our emphasis here was not on examining the long-time averages of brain synchronization but the dynamics of synchronization itself, expressed in a continual and fast changing pattern of functional couplings across network nodes. Our results indicate that these patterns are not randomly organized; rather they form a finite set or repertoire of network state families that consists of patterns that recur across time and are shared across individual participants.

One possible interpretation of the repertoire of network states is that these states represent patterns of synchronized neural activity distributed across the brain, i.e., the effects of true interactions between multiple neural sources. Alternative explanations include that these patterns were induced by intrinsic biases in the synchronization measure, artifacts of the clustering algorithm, or by volume conduction effects (e.g., Stam, 2005; Tognoli and Kelso, 2009). While biases in SL itself cannot be completely discounted, the measure has been tested against a number of canonical dynamical systems with tunable coupling parameters (e.g., Henon map) and it has been demonstrated that SL can sensitively detect slight variations in the coupling strength. Moreover, in a single simulation, SL distinguishes between periods of time when coupling was high versus periods of time when coupling was low. These results suggest that SL is capable of resolving synchronization patterns at a fine time scale. Furthermore, our results are strongest in frequency bands whose spectral content includes slow oscillations (i.e., Broadband, Theta, and Alpha). With low-frequency oscillations, fluctuations necessarily take place on a slower time scale than in Beta or Gamma ranges, and this supports the notion that SL can detect the slower-changing patterns of synchronization. It is also possible that SL depends critically on the choice of parameter values. Due to the dimensionality of the parameter space, it was impractical to consider the effect that all possible parameter values might have on the outcome of the SL routine. We note that it is possible that temporal parameters of the SL procedure and clustering methods settings can affect the observed time scales. However, the extent of this effect was not sufficiently investigated due to computational demands. Thus, the observed time scales of the network states and their durations may crucially depend on the SL parameters or clustering schema and may not reflect the corresponding scale of a neural “timekeeper.”

Volume conduction effects can give rise to synchronization between recording electrodes that are not driven by coordinated activity among distributed neural populations but instead generated from common dipole sources. A full analysis of possible volume conduction effects, e.g., through forward modeling of EEG signals and reconstruction of networks resulting from spatially distributed dipoles, is beyond the scope of this report. While a contribution to network synchrony from common dipole sources cannot be conclusively ruled out given the data, several observations suggest that such effects were not the sole generators of network states and state transitions. Correlations generated from single dipoles tend to be strongest between near-by (adjacent) electrodes (though this is not always the case, as the choice of reference electrode plays a role as well). However, in our data, strong and significant SL couplings arise across all distances. For example, spatially distributed edge communities that showed correlated state transitions (Figure 4C) and state families comprising numerous long-distance anterior-posterior and inter-hemispheric couplings (Figure 7) are difficult to reconcile with the expected spatial distribution of volume conduction effects. In addition, network state transitions were not associated with a global rise or fall of synchrony as might be expected if transitions were induced by intermittent activity from a deep common source. Instead, state transitions were largely uncorrelated with temporal variations in network density (Figure 3B). Taken together, these observations suggest that volume conduction alone does not account for the observed synchronization dynamics. A number of measures offer very conservative corrections to account for volume conduction, notably the phase-lag index proposed by Stam et al. (2007) and the phase-slope index proposed by Nolte et al. (2008). Both measures utilize relative phase a measure of coupling, which is a slightly less general measure of synchronization than SL. To effectively employ these measure would mean recasting our analyses in terms of this measure (or this measure and SL, simultaneously), which is beyond the scope of this study.

Our work is consistent with previous EEG/MEG studies of dynamic and nonstationary brain activity. Early work reported the existence of “EEG microstates,” brief episodes of stable spatial distributions of electric potentials lasting on the order of 100 ms (Lehmann et al., 1987; Lehmann, 1990), undergoing rapid transitions in the course of quasi- or metastable dynamics. EEG microstates can be classified into a small set of classes and they unfold across time in specific sequences that are thought to reflect the momentary fluctuations of mental processing. The durations of EEG microstates are distributed according to a power law (Van de Ville et al., 2010). We computed microstates for a small subsample of epochs and found that there was no clear mapping between microstates and network states, as defined in this report. This is somewhat unsurprising, as microstate detection is based on a clustering voltage distributions and the global field power (GFP) signal, both of which are independent of inter-channel coupling, which is the principle measure employed in this report. In MEG, broadband power time series show marked nonstationarity, with large power fluctuations that occur on a time scale of seconds (de Pasquale et al., 2010). These nonstationarities result in variable synchronization patterns, with intermittent epochs of high functional coupling that are interspersed by more weakly coherent episodes. Both EEG microstates and MEG nonstationarities are related to spontaneous signal fluctuations observed in resting-state fMRI. In simultaneous EEG/fMRI recordings EEG microstates correlate with BOLD activation patterns that resemble the signatures of specific resting-state networks (Britz et al., 2010; Musso et al., 2010). These findings raise the possibility that the stable and robust patterns described by long-time averages of BOLD fluctuations represent the accumulated effects of much faster, much more dynamic fluctuations in global network states. Our study suggests a novel neural correlate of these fluctuations in fast-changing networks of brain-wide synchronization.

The present study can be extended in several ways. First, it will be important to establish whether the rapid dynamics observed here at rest are maintained or suspended during task-evoked cognitive function. Prior studies indicate that functional brain networks rapidly reconfigure in response to task demands (Bassett et al., 2006) but it remains to be seen if the temporal dynamics of spontaneous network fluctuations are affected as well. Second, EEG recordings obtained at higher spatial resolution may be useful for determining more precisely the topographic distribution of variable network edges and of network modules across time. Finally, given data on disturbances in temporal coordination (Carroll et al., 2008) and synchronization (Brenner et al., 2003) in clinical condition such as schizophrenia, it may be fruitful to compare spatial and temporal characteristics of network dynamics between patient groups and healthy participants. These and other future studies are needed to clarify the association of synchronization dynamics with neural processes that underpin cognitive function.

### Conflict of interest statement

The authors declare that the research was conducted in the absence of any commercial or financial relationships that could be construed as a potential conflict of interest.
